# Unveiling atom-photon quasi-bound states in hybrid plasmonic-photonic cavity

**DOI:** 10.1515/nanoph-2022-0162

**Published:** 2022-06-09

**Authors:** Yu-Wei Lu, Wen-Jie Zhou, Yongyao Li, Runhua Li, Jing-Feng Liu, Lin Wu, Haishu Tan

**Affiliations:** School of Physics and Optoelectronic Engineering, Foshan University, Foshan 528000, China; Science, Mathematics and Technology (SMT), Singapore University of Technology and Design (SUTD), 8 Somapah Road 487372, Singapore, Singapore; Guangdong-Hong Kong-Macao Joint Laboratory for Intelligent Micro-Nano Optoelectronic Technology, Foshan University, Foshan 528000, China; School of Physics and Optoelectronics, South China University of Technology, Guangzhou 510641, China; College of Electronic Engineering, South China Agricultural University, Guangzhou 510642, China; Institute of High Performance Computing, Agency for Science, Technology, and Research (A*STAR), 1 Fusionopolis Way, #16-16 Connexis 138632, Singapore, Singapore

**Keywords:** atom-photon quasi-bound states, local density of states, plasmonic-photonic cavity

## Abstract

Dissipation, often associated with plasmons, leads to decoherence and is generally considered fatal for quantum nonlinearities and entanglement. Counterintuitively, by introducing a dissipative plasmonic nanoantenna into a typical cavity quantum electrodynamics (QED) system, we unveil the wide existence of the atom-photon quasi-bound state (qBS), a kind of exotic eigenstate with anomalously small decay, in the hybrid plasmonic-photonic cavity. To derive the analytical condition of atom-photon qBS, we formulate a quantized two-mode model of the local density of states by connecting the interacting uncoupled cavity modes to the macroscopic QED. With resonant plasmon-photon coupling, we showcase the single-atom qBS that improves the efficiency of single-photon generation over one order of magnitude; and the two-atom qBS that significantly enhances spontaneous entanglement generation compared with a bare photonic cavity. Notably, such single-atom and multi-atom qBS can be simultaneously accessed in realistic plasmonic-photonic cavities, providing a versatile platform for advanced quantum technologies, such as quantum light sources, quantum computation, and quantum information.

## 1 Introduction

With suppressed radiative leakage, single photons in a high-*Q* dielectric microcavity have the opportunity to interact repeatedly with a quantum emitter (QE), resulting in an enhanced coherent light–matter interaction and a strong coupling. Such strongly coupled microcavity-QE systems allow the on-chip manipulation, transmission, and storage of quantum states with high fidelity [1, 2]. However, their large physical volume (with at least one dimension on the order of the resonant wavelength) limits the further enhancement of the light–matter interaction in the traditional dielectric microcavity. Recently, the hybrid plasmonic-photonic cavity has emerged as a novel nanophotonic platform that can simultaneously exploit the advantages of both the low-loss microcavity and the large local field enhancement of plasmonic antenna [3–5]. In particular, when the microcavity is significantly red-detuned from the plasmonic antenna, the local density of states (LDOS), a fundamental quantity that governs the QE dynamics, manifests several-fold enhancement while the linewidth remains comparable to a bare microcavity [3, 4, 6], [7], [8]. This feature simultaneously enables high cooperativity of light–matter interaction and avoids undesirable dissipative losses. Accordingly, the red-detuned plasmonic-photonic cavity benefits various classical and quantum-optics applications, including biosensing [9, 10], nanolasers [11], optomechanics [12], strong light–matter interaction [13–15], and quantum light sources [16]. On the contrary, for the resonant plasmonic-photonic cavity (which is commonly characterized by much better field enhancements, mode volumes, and cooperativity), the LDOS is, however, strongly suppressed and dominated by the intrinsic ohmic loss of the plasmonic antenna, implying the weak coherent light–matter interaction in the near-resonance region.

**Figure 1:**
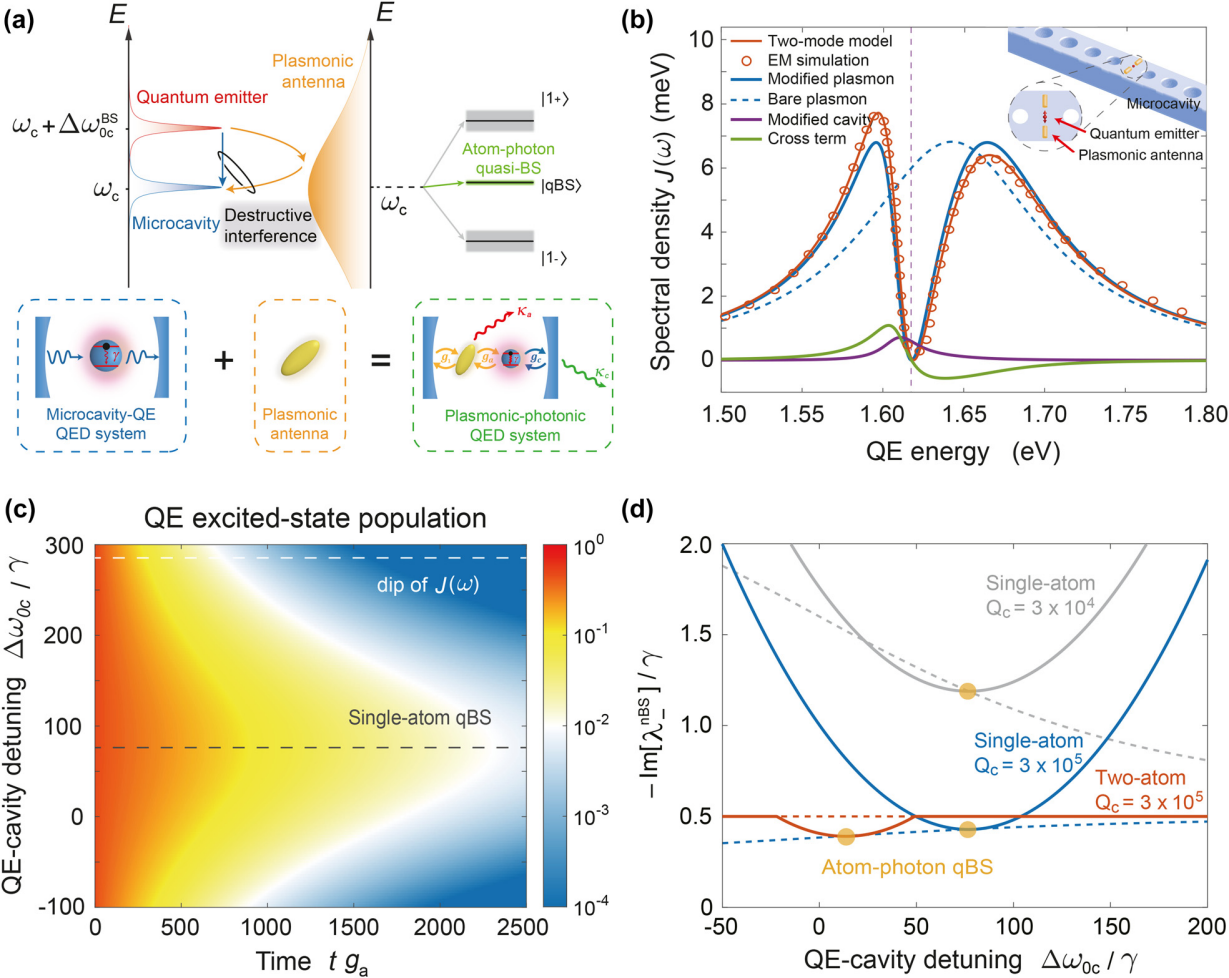
Atom-photon qBS in a hybrid plasmonic-photonic cavity QED system. (a) Concept diagram of atom-photon qBS in hybrid cavity. The atom-photon qBS occurs due to the destructive interference between two coupling channels belonging to QE and photonic microcavity, which do not need to overlap. The resultant eigenenergy spectrum at the single-excitation subspace has triplet sublevels. (b) Application of the two-mode model to calculate the spectral density *J*(*ω*) and identify different components in a sample hybrid cavity QED system [4], where a QE with dipole moment *μ* = 60 D at the gap center of a plasmonic antenna is placed on top of a dielectric nanobeam (inset). The vertical dashed line indicates the bare cavity resonance *ω*_c_ with decay rate *κ*_c_ = 5.4 μeV. (c) Relaxation dynamics of an initially excited QE (i.e., the color-map represents the excited-state population $\left\langle \sigma_{+}\sigma_{-}\right\rangle$ of QE) in the hybrid cavity as the function of QE-cavity detuning Δ*ω*_0c_, with QE’s decay rate *γ* = 10 μeV. The white and gray dashed lines indicate the dip of *J*(*ω*) and the optimal Δ*ω*_0c_ corresponding to single-atom qBS, respectively. (d) The imaginary part of the calculated eigenenergy corresponding to atom-photon qBS as a function of the Δ*ω*_0c_ for single-atom and two-atom cases at the different microcavity quality factors *Q*_c_, while those of bare microcavity are also shown for comparison (dashed lines with the same color). The yellow dots label the conditions of the atom-photon qBS.

Despite the inevitable large nonradiative dissipation in the resonant plasmon-photon coupling, in this work, we will show that introducing a resonant plasmonic antenna allows for forming an exotic eigenstate whose decay is equal to or even smaller than the original cavity quantum electrodynamics (QED) system. The underlying physical mechanism of this anomalously small decay is analogous to the accidental bound state in the continuum (BIC), which is a localized resonance with an infinite lifetime inside the continuum achieved through parameter tuning [17]. BIC was originally proposed in quantum mechanics when studying the one-dimensional Schrödinger equation [18] and the interference of two resonances associated with different channels [19], but it also flourished in many fields of physics, including the metamaterial and quantum material [20, 21], quantum optics [22–25], and acoustic systems [26, 27]. Particularly, in recent years, BIC has arisen as a new strategy to trap and guide the optical waves in various photonic systems [28–33]. Beyond these classical scattering and absorption phenomena, the quantum effects in BIC quantum systems play an important role in various quantum applications, such as the manipulation of quantum light [21, 24, 25] and the long-distance photon propagation and quantum entanglement in waveguide-QED systems [34–36]. In this work, based on the plasmonic-photonic cavity, we reveal the existence of quasi-bound states (qBS) for the atom–photon interaction. As illustrated in Figure 1a, the broadband plasmon resonance from the plasmonic antenna acts as the continuum in the plasmonic-photonic QED system. The atom–photon qBS appears due to the destructive interference of the “direct” and “plasmon-mediated” couplings between the microcavity and the QE. The resultant eigenenergy spectrum has triplet eigenenergy levels at the one-excitation subspace. The decay of lower and upper bands is about the quart linewidth of plasmon resonance, while the middle one with anomalously small decay defines the atom-photon qBS.

Obtaining the concrete condition of atom-photon qBS requires identifying different coupling pathways. However, the quantized treatment of the plasmonic-photonic cavity is complicated due to the dispersive and absorbing characteristics of materials, and the mutual coupling of two components. Two powerful and efficient methods, the quasinormal mode [8, 37] and the quantized pseudomode [3, 7, 38], have been applied to study the classical and quantum properties of the plasmonic-photonic cavity. Inspired by them and to elucidate the role of different coupling channels in forming the atom-photon qBS and determine the critical system parameters, we develop a simple and intuitive two-mode quantization model for the LDOS of the plasmonic-photonic cavity, where the mode parameters and the coupling rates with QE are obtained from bare components, leaving only the mode coupling determined by the simple curve fitting. We further demonstrate that the small decay of atom-photon qBS makes the plasmonic-photonic cavity advantageous in enhancing single-photon generation and the spontaneous entanglement generation (SEG) in the regime of on- and near-resonance plasmon-photon coupling.

## 2 Results and discussion

### 2.1 Two-mode model for LDOS of hybrid cavity

We consider a dipolar plasmonic antenna, where the higher-order modes are well separated from the dipolar plasmonic mode, then the hybrid plasmonic-photonic cavity can be treated as two coupled Lorentzian cavities. The full Hamiltonian of a two-level QE interacting with hybrid cavity reads *H* = *H*_0_ + *H*_I_, including the free Hamiltonian $H_{\text{0}}=\left(\omega_{\text{0}}-i\gamma /2\right)\sigma_{+}\sigma_{-}+\left(\omega_{\text{a}}-i\kappa_{\text{a}}/2\right)a^{†}a+\left(\omega_{\text{c}}-i\kappa_{\text{c}}/2\right)c^{†}c$ and the interaction Hamiltonian $H_{\text{I}}=g_{\text{a}}\left(a^{†}\sigma_{-}+\sigma_{+}a\right)+g_{\text{c}}\left(c^{†}\sigma_{-}+\sigma_{+}c\right)-g_{1}\left(a^{†}c+c^{†}a\right)$ , where the decay of each component is introduced via quantum-jump approach and well approximately by the jump-free part [39], since we consider the single and weak excitation in this work. Here, *a* (or *a* ^†^) and *c* (or *c* ^†^) represent the bosonic annihilation (or creation) operators for the dipolar plasmonic mode and the microcavity mode, with resonance frequencies *ω*_a_ and *ω*_c_, and decays *κ*_a_ and *κ*_c_, respectively; *σ*_−_ = |*g*⟩⟨*e*| (or *σ*_+_ = |*e*⟩⟨*g*|) denotes the lowing (or raising) operator of QE with *ω*_0_ and *γ* the corresponding transition frequency and decay of QE; *g*_a_, *g*_c_ and *g*_1_ are the coupling rates for QE-plasmon, QE-photon and plasmon-photon interaction, respectively. The spectral density of the hybrid cavity is expressed as: $J\left(\omega \right)=\int_{-\infty }^{+\infty }d\tau \Lambda \left(\tau \right){\text{e}}^{\text{i}\omega t}$ , with $\Lambda \left(\tau \right)=\left\langle \left[g_{\text{a}}a\left(\tau \right)+g_{\text{c}}c\left(\tau \right)\right]\left[g_{\text{a}}a^{†}\left(0\right)+g_{\text{c}}c^{†}\left(0\right)\right]\right\rangle$ [40, 41], which can be analytically obtained by calculating the environmental correlation functions from full Hamiltonian *H*, and separated into three parts (see details in Supporting Information S1):

(1) J(ω)=ReJa(ω)+Jc(ω)+Ja c(ω),

with

(2) $$J_{X}\left(\omega \right)=ig_{\text{X}}^{2}\chi_{\text{X}}\left(\omega \right){\left[1-g_{1}^{2}\chi_{\text{a}}\left(\omega \right)\chi_{\text{c}}\left(\omega \right)\right]}^{-1},$$

(3) $$J_{\text{ac}}\left(\omega \right)=i2g_{\text{a}}g_{\text{c}}g_{1}\chi_{\text{a}}\left(\omega \right)\chi_{\text{c}}\left(\omega \right){\left[1-g_{1}^{2}\chi_{\text{a}}\left(\omega \right)\chi_{\text{c}}\left(\omega \right)\right]}^{-1},$$

where $\chi_{\text{X}}\left(\omega \right)={\left[\left(\omega -\omega_{\text{X}}\right)+i\kappa_{\text{X}}/2\right]}^{-1}$ for *X* = *a*, *c* is the polarizability of bare components. *J*_a_(*ω*) and *J*_c_(*ω*) represent the modified plasmon and cavity response, respectively, and *J*_ac_(*ω*) is the crossing interference term. Therefore, *J*(*ω*) can be generated via the parameters of bare cavity modes and their coupling rate. The normalized LDOS, i.e., Purcell factor, is related to the spectral density through the relationship *P*(*ω*) = *J*(*ω*)/*J*_0_(*ω*), where *J*_0_(*ω*) = *ω* ^3^ *μ* ^2^/6*π* ^2^ *ℏɛ*_0_ *c* ^3^ is the spectral density in free space (*ɛ*_0_ the vacuum permittivity and *c* the speed of light) for a QE with dipole moment *μ*.

To validate our model, we take a recently reported hybrid cavity [4] as an example, i.e. a gold dimer on nanobeam cavity shown as the inset of Figure 1b, and parametrize its spectral density. For a QE with *μ* = 60 D, we obtain the parameters $\left(\omega_{\text{a}},\kappa_{\text{a}},g_{\text{a}}\right)=$ (1.643 eV, 0.136 eV, 21 meV) for the bare plasmonic antenna and $\left(\omega_{\text{c}},\kappa_{\text{c}},g_{\text{c}}\right)$ = (1.618 eV, 5.4 μeV, 0.94 meV) for the bare nanobeam microcavity (with quality factor *Q*_c_ ≡ *ω*_c_/*κ*_c_ = 3 × 10^5^). Such hybrid cavity can be considered as resonant coupling since the plasmon–photon detuning Δ*ω*_ac_ = *ω*_a_ − *ω*_c_ ≈ 0.18*κ*_a_. Subsequently, the mode coupling *g*_1_ can be evaluated by a simple curve fitting using Eqs (1)–(3), and the optimal value is found to be *g*_1_ = 32 meV. The corresponding LDOS generated by our two-mode model is plotted in Figure 1b, which matches well with the classical electromagnetic (EM) simulation. More essentially, our model breaks down the spectral density into three terms that correspond to different coupling pathways. As shown in Figure 1b, the contribution of the plasmon-modified cavity response (purple) can be neglected compared to the cavity-modified plasmon response (blue), which dominates the spectral density. Around the cavity resonance *ω*_c_ = 1.618 eV, the interference cross term (green) changes its sign and leads to an asymmetric spectral density. Meanwhile, the cavity-modified plasmon response (blue), and hence the spectral density (red), are strongly suppressed, where a dip can be clearly observed due to the destructive interference between the nanobeam cavity and the plasmonic antenna. This leads to the slower relaxation (longer lifetime) of an excited QE with transition frequency lying in this region when the QE-cavity detuning Δ*ω*_0c_ = *ω*_0_ − *ω*_c_ is close to zero, as detailed in Figure 1c where we normalize Δ*ω*_0c_ to the decay rate *γ* = 10 μeV of QE. The slowest relaxation occurs when QE is slightly blue-detuned ( ∼0.76 meV) from the nanobeam cavity, neither on resonance nor at the dip of *J*(*ω*) but between them. This slowest relaxation condition, relevant to the atom-photon quasi-bound states (*e*.*g*., single-atom qBS in this case), will be explained in more detail in the following. It should be emphasized that the microcavity resonance frequency *ω*_c_ = 1.618 eV and the QE’s decay rate *γ* = 10 μeV are kept constant throughout this work as the reference values.

### 2.2 Generalized analytical condition of *N*-atom-photon qBS

Based on our quantized two-mode model, we can unravel the underlying mechanism and generalize the plasmonic-photonic QED system to the *N*-atom case. The dynamics of the plasmonic-photonic QED system follows the quantum master equation (QME):

(4) $$\frac{\partial \rho }{\partial t}=i\left[\rho ,H_{\text{M}}\right]+\frac{\kappa_{\text{a}}}{2}L_{\text{a}}\left(\rho \right)+\frac{\kappa_{\text{c}}}{2}L_{\text{c}}\left(\rho \right)+\underset{i}{\sum }\frac{\gamma }{2}L_{\sigma_{-}^{\left(i\right)}}\left(\rho \right),$$

where $L_{\hat{o}}\left(\rho \right)=2\hat{o}\rho {\hat{o}}^{†}-{\hat{o}}^{†}\hat{o}\rho -\rho {\hat{o}}^{†}\hat{o}$ is the Liouvillian superoperator with $\hat{o}$ being an operator. The corresponding multi-QE Hamiltonian *H*_M_ reads:

(5) $$\begin{array}{ll} H_{\text{M}} & =\omega_{\text{a}}a^{†}a+\omega_{\text{c}}c^{†}c+\underset{i}{\sum }\omega_{0}\sigma_{+}^{\left(i\right)}\sigma_{-}^{\left(i\right)}\\ & \;+\underset{i}{\sum }g_{\text{a}}^{\left(i\right)}\left(a^{†}\sigma_{-}^{\left(i\right)}+\sigma_{+}^{\left(i\right)}a\right)\\ & \;+\underset{i}{\sum }g_{\text{c}}\left(c^{†}\sigma_{-}^{\left(i\right)}+\sigma_{+}^{\left(i\right)}c\right), \end{array}$$

where we have made the assumption of identical QEs having different coupling strengths with the plasmonic antenna, considering that the EM field distribution of plasmonic mode can drastically vary within a few nanometers from the antenna. In the case of single excitation or the system is driven by a weak coherent field, the system dynamics can be well described by the jump-free part of $L_{\hat{o}}\left(\rho \right)$ . We can derive a non-Hermitian Hamiltonian $H^{\prime }=H_{\text{0}}^{\prime }+H_{\text{I}}^{\prime }$ from the above QME, with the free Hamiltonian $H_{\text{0}}^{\prime }=\sum_{i}\left(\omega_{\text{0}}-i\gamma /2\right)\sigma_{+}^{\left(i\right)}\sigma_{-}^{\left(i\right)}+\left(\omega_{\text{a}}-i\kappa_{\text{a}}/2\right)a^{†}a+(\omega_{\text{c}}-i\kappa_{\text{c}}/2)c^{†}c$ and the interaction Hamiltonian $H_{\text{I}}^{\prime }=\sum_{i}g_{\text{a}}^{\left(i\right)}\left(a^{†}\sigma_{-}^{\left(i\right)}+\sigma_{+}^{\left(i\right)}a\right)+\sum_{i}g_{\text{c}}\left(c^{†}\sigma_{-}^{\left(i\right)}+\sigma_{+}^{\left(i\right)}c\right)-g_{1}(a^{†}c+c^{†}a)$ . Rewriting the Hamiltonian *H*′ in the one-excitation subspace in matrix form (see details in Supporting Information S2), in the limit of *κ*_a_ ≫ *κ*_c_, *γ*, which is realistic for plasmonic-photonic cavity, we find the solution for a purely real eigenenergy if the QE-cavity detuning Δ*ω*_0c_ meets the following requirement:

(6) $$\Delta \omega_{\text{0c}}^{nBS}=g_{\text{c}}\left(\frac{Ng_{1}}{\underset{i}{\sum }g_{\text{a}}^{\left(i\right)}}-\frac{\underset{i}{\sum }g_{\text{a}}^{\left(i\right)}}{g_{1}}\right).$$

At this point, one of eigenenergies is purely real, which is $\lambda_{-}^{nBS}=\omega_{\text{c}}+Ng_{1}g_{\text{c}}/\sum_{i}g_{\text{a}}^{\left(i\right)}$ , with *N* being the number of QEs. For single-atom case with *N* = 1, the above condition is $\Delta \omega_{\text{0c}}^{\text{BS}}=g_{\text{c}}\left(g_{1}/g_{\text{a}}-g_{\text{a}}/g_{1}\right)$ , which takes the same form as the condition of Friedrich-Wintgen BIC [17, 19], and thus we call this eigenstate as the single-atom-photon qBS. Note that the genuine bound state can hardly form due to the existence of decays *κ*_c_ and *γ*. In Figure 1c, this single-atom qBS is indicated by a dashed gray line, which coincides with the slowest relaxation of QE.

To make things more general, we plot the eigenenergy corresponding to the single-atom and two-atom qBS as a function of QE-cavity detuning Δ*ω*_0c_ in Figure 1d, where the imaginary part of eigenenergy (i.e., decay) reaches the minimum (highlighted using yellow dots) at the qBS condition given by Eq. (6) in both cases. For the single-atom case, it is evident that the decay of single-atom qBS (solid lines) is generally smaller than that of the atom-like branch of bare microcavity (dashed lines) at the qBS condition. Even if the qBS condition is not exactly fulfilled, this anomalously small decay can persist over a finite frequency range around $\Delta \omega_{\text{0c}}^{nBS}$ (gray lines). However, it is harder to recognize this feature for a high-*Q* cavity (blue lines). For the two-atom case (orange lines), the decay at qBS is smaller than that of the two-atom dark state in a bare microcavity (i.e., *γ*/2), while it recovers back to *γ*/2 when far from the qBS condition. In short, it is instructive to derive the generalized analytical condition of *N*-atom-photon qBS using Eq. (6) for any practical application, and we will show its applicability to predict the optimal conditions for quantum-optics applications in the latter part of the work.

### 2.3 Quantum-optics applications of atom-photon qBS

The feature of small decay makes atom-photon qBS beneficial for many quantum-optics applications. Here, we will showcase two examples: single-atom qBS for improving the efficiency of single-photon generation using conventional photon blockade (CPB) and two-atom qBS for enhancing the spontaneous entanglement generation (SEG) between two QEs.

#### 2.3.1 Single-atom qBS for single-photon generation

Single-photon generation via conventional photon blockade (CPB) utilizes the anharmonic energy levels to block the absorption of the second photon. The zero-time-delay correlation function *g* ^(2)^(0) < 1, i.e., photon antibunching, signs the occurrence of single-photon blockade. As it takes place when the system is driven at the frequency of one of the eigenstates at the single-excitation subspace [42], the feature of small decay makes single-atom qBS particularly beneficial for improving the photon intensity, and hence the efficiency of single-photon generation using CPB. To investigate the performance of single-photon generation, a weak coherent field is applied to the system by implementing a driving Hamiltonian *H*_drive_ in QME in Eq. (4), which takes the form of $H_{\text{drive}}=\Omega \left({\text{e}}^{-\text{i}\omega_{\text{L}}t}\sigma_{+}+\sigma_{-}{\text{e}}^{\text{i}\omega_{\text{L}}t}\right)$ for QE drive and $H_{\text{drive }}=\Omega \left({\text{e}}^{-\text{i}\omega_{\text{L}}t}c^{†}+c{\text{e}}^{\text{i}\omega_{\text{L}}t}\right)$ for cavity drive, where Ω and *ω*_L_ are the intensity and the laser frequency of driving field, respectively. The numerical calculation details for the single-photon intensity $\left(I_{\text{c}}=\left\langle c^{†}c\right\rangle \right)$ and the purity $\left(g^{\left(2\right)}\left(0\right)=\left\langle c^{†}c^{†}cc\right\rangle /I_{\text{c}}^{2}\right)$ can be found in Supporting Information S3.

**Figure 2:**
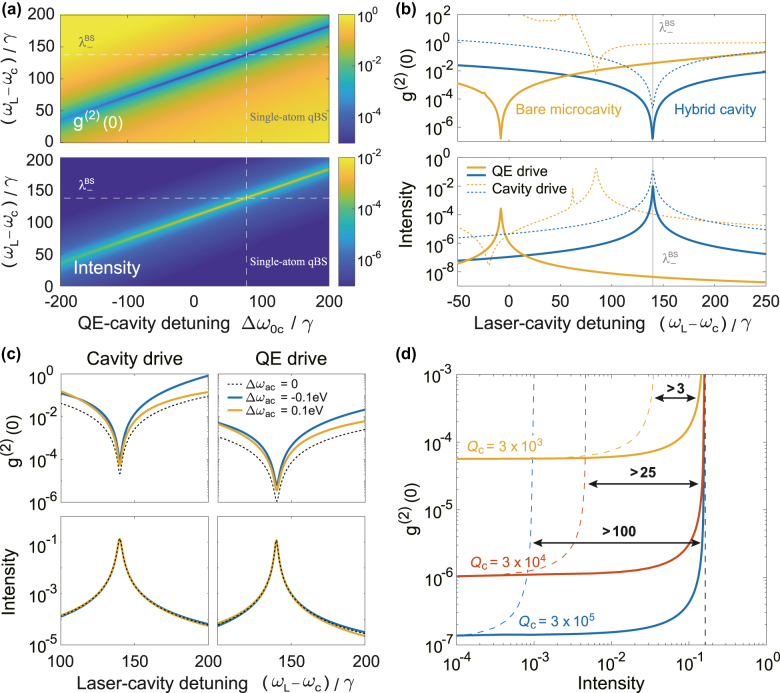
Single-atom qBS for single-photon generation. (a) The zero-time-delay correlation function *g* ^(2)^(0) (upper panel) and intensity (lower panel) in the cavity-driven case as the function of QE-cavity detuning Δ*ω*_0c_ and the laser-cavity detuning, with *ω*_L_ the laser frequency. The vertical and horizontal dashed lines correspond to the optimal Δ*ω*_0c_ and the eigenenergy of single-atom qBS, respectively. (b) The *g* ^(2)^(0) (upper panel) and intensity (lower panel) versus laser-cavity detuning for bare microcavity (yellow lines) and hybrid cavity (blue lines) with single-atom qBS for QE drive (solid lines) and cavity drive (dashed lines). The light gray lines indicate the location of $\lambda_{-}^{\text{BS}}$ . The driving strength is *Ω* = 0.1*γ* for QE drive, and *Ω* = *γ* for cavity drive. (c) The *g* ^(2)^(0) (upper panel) and intensity (lower panel) versus laser-cavity detuning for hybrid cavity with three different plasmon-photon detuning Δ*ω*_ac_. The driving strength is *Ω* = *γ*. (d) The intensity-purity curves for the hybrid cavity (solid lines) and bare microcavity (dashed lines) with different quality factors under QE drive. Here, $\Delta \omega_{\text{0c}}=\Delta \omega_{\text{0c}}^{\text{BS}}$ for hybrid cavity, while Δ*ω*_0*c*_ of bare microcavity are 1960*γ*, 1000*γ*, and 320*γ* for *Q*_c_ = 3 × 10^3^, *Q*_c_ = 3 × 10^4^ and *Q*_c_ = 3 × 10^5^, respectively. The vertical black dashed line indicates the achievable maximum intensity of hybrid cavity. Parameters of the hybrid cavity are kept the same as those in Figure 1 unless specially noted, where the microcavity resonance frequency *ω*_c_ = 1.618 eV and the QE’s decay rate *γ* = 10 μeV are kept constant throughout this work as the reference values.

Figure 2a shows the calculated zero-time-delay correlation function *g* ^(2)^(0) and single-photon intensity *I*_c_ of the nanobeam microcavity for the cavity-driven case by varying pump frequency *ω*_L_ and QE-cavity detuning Δ*ω*_0c_. We can see that the best single-photon purity and highest intensity are simultaneously achieved at single-atom qBS. We then compare the single-photon performance of hybrid cavity with single-atom qBS and bare nanobeam microcavity in Figure 2b. It shows that when achieving the same single-photon purity, *g* ^(2)^(0) = 10^−7^ with QE drive, the intensity of hybrid cavity (solid blue line) is boosted more than 36-fold compared to a bare nanobeam microcavity (orange solid line). While driving the cavity with a stronger pump (dashed lines), the single-photon purity *g* ^(2)^(0) can be improved over two orders of magnitude when achieving similar intensity.

On the other hand, it is worth noting that though the resonant plasmon-photon coupling manifests the best single-photon performance, the moderate plasmon-photon detuning does not significantly degrade the single-photon purity and intensity. As indicated in Figure 2c, the increase of minimum *g* ^(2)^(0) is within one order of magnitude with plasmon-photon detuning Δ*ω*_ac_ = *ω*_a_ − *ω*_c_ = ±0.1 eV, compared to the resonant case; whereas the variation of intensity is negligible. Furthermore, we evaluate from Figure 2a that both the intensity and purity decrease less than 10% with QE-microcavity detuning $\left|\Delta \omega_{\text{0c}}-\Delta \omega_{\text{0c}}^{nBS}\right|<10\gamma$ . Therefore, the single-atom qBS indeed makes the plasmonic-photonic cavity as a robust platform for high-efficiency single-photon source.

We further compare the intensity-purity curves for hybrid cavity and bare microcavity, obtained by increasing the driving strength for different microcavity quality factor *Q*_c_ under QE drive condition, as illustrated in Figure 2d. There is clearly an upper bound for the intensity, which is independent of *Q*_c_ (but mainly limited by QE-photon coupling rate *g*_c_). A high *Q*_c_ improves the single-photon purity with significantly smaller *g* ^(2)^(0). Compared to a bare microcavity (dashed lines) achieving the same *g* ^(2)^(0) in the weak pump limit $\left(Q_{\text{c}}=3\times 10^{5}\right)$ , the hybrid cavity (solid lines) demonstrates a hundredfold enhancement of the maximum intensity. Such enhancement drops from 100 to 25 as *Q*_c_ decreases from 3 × 10^5^ to 3 × 10^4^. These results suggest that the figure of merit of the microcavity is critical for the efficiency enhancement of single-photon blockade with single-atom qBS.

The single-photon generation discussed in this work is based on the conventional photon blockade (CPB) that utilizes the anharmonicity of the discrete level structure. An alternative route to generate a single photon from coherent light is to induce the destructive interference between all possible transition pathways for the two-photon state of the microcavity, called unconventional photon blockade (UPB) [42]. The intensity (efficiency) of UPB is generally lower than CPB by orders of magnitude (e.g., in a QE-microcavity system [43]) as CPB occurs at one of the peaks of intensity while UPB does not. For example, the intensity of UPB in a coupled quantum-dot-cavity system is 0.004 for *g* ^(2)^(0) ≈ 0.005 [44], while the intensity of CPB in our system is roughly 0.2 for $g^{\left(2\right)}\left(0\right)=0.001\;\left(Q_{c}=3\times 10^{3}\right)$ , as indicated in Figure 2d. The single-atom qBS combined with CPB is a promising scheme for building a high-efficiency quantum light source.

#### 2.3.2 Two-atom qBS for spontaneous entanglement generation

The creation of entangled states between the qubits is a key task of quantum computation and quantum information processing [45]. The entanglement is commonly measured by the concurrence *C*(*t*) [46], which in our system is given by the simple expression $C\left(t\right)=2\left|C_{\text{eg}}\left(t\right)C_{\text{ge}}^{*}\left(t\right)\right|$ , with *C*_eg_(*t*) and *C*_ge_(*t*) the probability amplitudes of two single-excitation states that one QE is in the excited state while the other is in the ground state at any point of time. To calculate *C*(*t*), we set one QE initially in the excited state and the other in the ground state and allow the two QEs to evolve spontaneously into an entangled state. During the evolution, we can identify the achievable maximal value of concurrence, i.e., max[*C*(*t*)]. The larger the value of max[*C*(*t*)], the better the entanglement between two QEs is. Here, we show that the two-atom qBS can enhance the spontaneous entanglement generation (SEG) between two QEs, where our generalized analysis of *N*-atom-photon qBS is applicable with *N* = 2.

**Figure 3:**
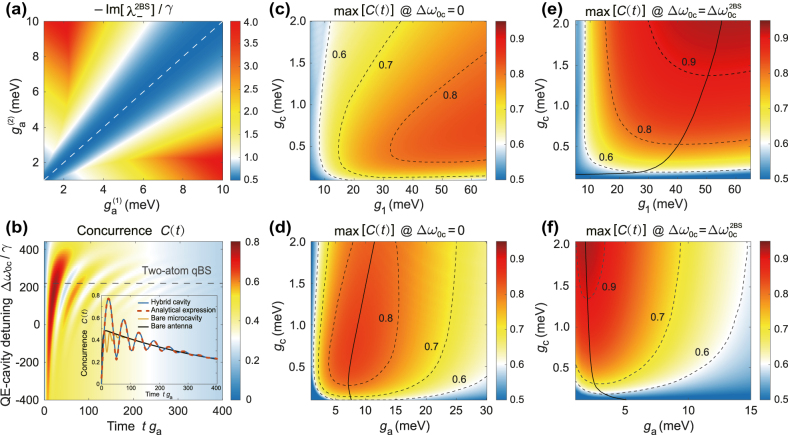
Two-atom qBS for spontaneous entanglement generation (SEG). (a) The imaginary part of the eigenenergy of two-atom qBS as the function of QE-plasmon coupling rates $g_{\text{a}}^{\left(1\right)}$ and $g_{\text{a}}^{\left(2\right)}$ , normalized by the constant QE’s decay rate *γ* = 10 μeV. The white dashed line traces the minimum decay. (b) Concurrence of SEG as the function of Δ*ω*_0c_. Inset: Comparison of the concurrence of hybrid cavity with an approximate analytical expression. Concurrence of bare microcavity and plasmonic antenna are also shown for comparison. (c) and (d) Maximum concurrence max[*C*(*t*)] as the function of QE-photon coupling rate *g*_c_ and plasmon-photon coupling rate *g*_1_, QE-photon coupling rate *g*_c_ and QE-plasmon coupling rate *g*_a_, respectively, in the condition of resonant coupling Δ*ω*_0c_ = 0. (e) and (f) are the same as (b) and (c) but for the case of two-atom qBS with $\Delta \omega_{\text{0c}}=\Delta \omega_{\text{0c}}^{2\text{BS}}$ . The black solid lines trace the maximum concurrence with respect to the QE-photon coupling rate *g*_*c*_. The parameters of the resonant plasmon-photon hybrid cavity are *ω*_a_ = *ω*_c_ = 1.618 eV, *g*_a_ = 5 meV, *g*_c_ = 0.6 meV, *g*_1_ = 20 meV, *κ*_a_ = 0.12 eV and *κ*_c_ = 0.5 meV (*Q*_c_ ∼ 3000). The numerical calculations of concurrence are performed using QuTip [47], with system’s initial condition: one QE is in the excited state and the other is in the ground state, while the cavity fields are in the vacuum state.

We first study the impact of different QE-plasmon couplings $g_{\text{a}}^{\left(i\right)}$ (*i* represents different QEs) on the decay of two-atom qBS in Figure 3a and find that $g_{\text{a}}^{\left(1\right)}=g_{\text{a}}^{\left(2\right)}$ is the optimal condition for minimum decay, which is slightly smaller than *γ*/2 and approaches *γ*/2 as the coupling rate of QE-plasmon interaction increases. Therefore, we only consider the case of $g_{\text{a}}^{\left(1\right)}=g_{\text{a}}^{\left(2\right)}$ in the following discussions. We then plot the concurrence *C*(*t*) of dynamical entanglement generation between an initially excited QE and a ground state QE as a function of the QE-cavity detuning in Figure 3b. Evidently, the *C*(*t*) demonstrates oscillating patterns around the two-atom qBS, where the most persistent oscillation occurs exactly at the two-atom qBS (highlighted by the dashed line). Taking a closer look at the inset, the max[*C*(*t*)] of the hybrid cavity is ∼0.77 , while those of the bare components are <0.48 .

The oscillating behavior of *C*(*t*) originates from the dissipative atom-photon interaction mediated by the plasmonic antenna, but the damping of oscillation is determined by the decay of eigenenergies and thus related to the two-atom qBS. This can be better understood from an approximate analytical expression of *C*(*t*) (see detailed derivation in Supporting Information S4):

(7) C(t)≈12e−γ−tκ−iΔω0c+u24|u|2−e−γt−ie−γ2te−γ−2tκRe[u]|u|2+1×sinImu−2γp+Δω0c2t,

with γ−=κ+γ/2−Reu−2γp , $u=\sqrt{-8g_{\text{ca}}g_{\text{ac}}+{\left[-i\Delta \omega_{\text{0c}}+\left(\kappa -2\gamma_{\text{p}}-\gamma /2\right)\right]}^{2}}$ , and *κ* = *κ*_c_/2 + *κ*_p_. Here, $\kappa_{\text{p}}=2g_{1}^{2}/\kappa_{\text{a}}$ and $\gamma_{\text{p}}=2g_{\text{a}}^{2}/\left(-i2\Delta \omega_{\text{0c}}+\kappa_{\text{a}}\right)$ are the plasmon-induced cavity and QE decays, respectively; *g*_ca_ = *g*_c_ + *i*2*g*_a_ *g*_1_/*κ*_a_ and $g_{\text{ac}}=g_{\text{c}}+i2g_{\text{a}}g_{1}/\left(-i2\Delta \omega_{\text{0c}}+\kappa_{\text{a}}\right)$ are the plasmon-mediated cavity-to-QE and QE-to-cavity couplings, respectively. Equation (7) is deduced from the effective Hamiltonian $H_{\text{eff}}=\sum_{i,j}\left[\left(\omega_{\text{c}}+\Delta \omega_{\text{0c}}-i\gamma /2\right)\delta_{ij}-i\gamma_{\text{p}}\right]\sigma_{+}^{\left(i\right)}\sigma_{-}^{\left(j\right)}+(\omega_{\text{c}}-i\kappa /2)c^{†}c+\sum_{i}\left(g_{\text{ac}}c^{†}\sigma_{-}^{\left(i\right)}+g_{\text{ca}}\sigma_{+}^{\left(i\right)}c\right)$ , obtained by adiabatically eliminating the plasmon operators. It can be seen from the inset of Figure 3b that our analytical expression Eq. (7) accords quite well with the numerical solution. Upon further analysis of Eq. (7) for the resonant condition (Δ*ω*_0*c*_ = 0), the *C*(*t*) oscillates due to Imu−2γp≠0 , which results from the co-existence of the coherent and the dissipative components in the effective atom-photon interaction for the case of hybrid cavity. Another point to note from Eq. (7), the parameter *γ*_−_ (corresponding to the damping of oscillations since *γ*_−_ ≫ *γ*) plays an essential role in determining the longtime dynamics of concurrence. In Figure 4 of Supporting Information, we show that *γ*_−_ reaches the minimum at two-atom qBS. It explains why two-atom qBS corresponds to the most persistent oscillations of *C*(*t*).

The competing coherent and dissipative interaction combined with two-atom qBS enables higher concurrence of SEG in the plasmonic-photonic cavity. Figure 3c and e) compare the maximum concurrence (max[*C*(*t*)]) of SEG in the hybrid cavity for resonant QE-cavity coupling condition and two-atom qBS condition, respectively, as the function of QE-photon coupling *g*_c_ and plasmon-photon coupling *g*_1_. For the resonant case in Figure 3c, larger plasmon-photon coupling *g*_1_ is beneficial for higher concurrence. On the other hand, there is an optimal *g*_1_ for the case of two-atom qBS, see solid black line in Figure 3e. Clearly, the two-atom qBS condition improves the maximum concurrence from 0.84 to 0.95 and meanwhile lowers the requirement of *g*_1_ for achieving the same concurrence. This advantage is more prominent with strong QE-photon coupling *g*_c_. For example, with *g*_c_ = 2 meV, a moderate plasmon–photon coupling *g*_1_ = 20–40 meV can increase the concurrence by ∼30% . Similarly, Figure 3d and f illustrates the dependence of the concurrence on QE-photon coupling *g*_c_ and QE-plasmon coupling *g*_a_. The optimal QE-plasmon coupling *g*_a_ to reach an even higher maximum concurrence at the two-atom qBS condition in Figure 3f is smaller than that for the resonant QE-cavity coupling condition in Figure 3d. These features will enhance the SEG and benefit the entanglement transport via microcavity.

To further understand the SEG in hybrid cavity, we calculate the fidelity $F_{\pm }=\left\langle \Psi_{\pm }|\rho |\Psi_{\pm }\right\rangle$ and show in Figure 5 of Supporting Information, with |Ψ_+_⟩ and |Ψ_−_⟩ being the even and odd Bell states, respectively. The results indicate that the created entangled state is the odd Bell state, and $\max\left[F_{-}\right]\approx \max\left[C\left(t\right)\right]$ for high concurrence. Compared to the recently reported SEG scheme based on the chiral waveguide $\left(\max\left[F_{-}\right]=\max\left[C\left(t\right)\right]=0.92\right)$ [48], which is promising for constructing the quantum networks, our system can achieve a comparable concurrence and fidelity of 0.95. This value is also higher than SEG of both the individual plasmonic antenna (∼0.87) [49] and the single-mode microcavity (∼0.89) [50]. Furthermore, it is important to note that the underlying physical mechanism for our atom-photon qBS is fundamentally different from the dissipation-induced stationary entanglement [50], although the plasmonic antenna plays the role of dissipative environment (the continuum). While the dissipation-induced stationary entanglement is attributed to the anti-symmetric state of two QEs decoupled from the cavity due to the dipole–dipole interaction, our atom-photon qBS results from the destructive interference of two coupling pathways. Still, placing the QEs inside the gap center of the plasmonic antenna and utilizing the dipole–dipole interaction makes it possible to realize the stationary entanglement in our setup.

#### Applicability of analytical condition $\Delta \omega_{\text{0c}}^{n\text{B}\text{S}}$

2.3.3

**Figure 4:**
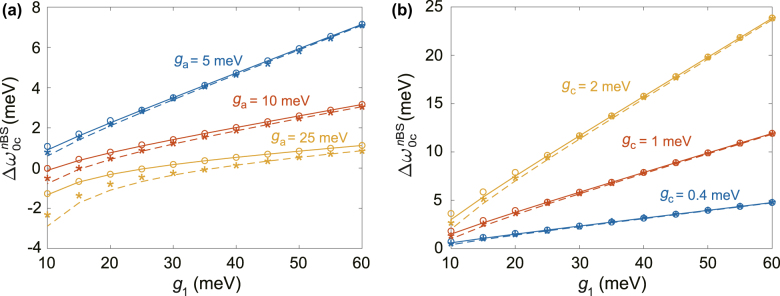
Applicability of analytical condition $\Delta \omega_{\text{0c}}^{nBS}$ to predict the optimal conditions for quantum-optics applications. Comparison of $\Delta \omega_{\text{0c}}^{\text{BS}}$ (solid lines) and the optimal QE-cavity detuning Δ*ω*_0c_ corresponding to the single-photon blockade with highest efficiency (hollow circles), as well as $\Delta \omega_{\text{0c}}^{2BS}$ (dashed lines) and the optimal Δ*ω*_0c_ for maximum concurrence in the longtime limit (stars) for different cases of **(a)** QE-plasmon coupling rate *g*_a_ and (b) QE-photon coupling rate *g*_c_, as a function of plasmon-photon coupling rate *g*_1_. All other parameters are kept the same as those in Figure 3 unless mentioned otherwise.

Up to this point, we have demonstrated two quantum optics applications of qBS and provided a handy formula for the condition of atom-photon qBS $\Delta \omega_{\text{0c}}^{nBS}$ in Eq. (6) to guide the design of experiment. However, we have neglected the decays (*κ*_c_ and *γ*) in deriving our analytical condition Eq. (6), which may influence its applicability. Next in Figure 4, we will investigate the robustness of $\Delta \omega_{\text{0c}}^{nBS}$ to accurately predict the optimal condition for single-photon blockade and SEG, by comparing it with the optimal Δ*ω*_0c_ (hollow circles for single-photon blockade and stars for SEG) obtained using various coupling parameters and taking into account the noise values *γ* = 10 μeV, *κ*_a_ = 0.12 eV, and *κ*_c_ = 0.5 meV. We can see that $\Delta \omega_{\text{0c}}^{nBS}$ generally shows good agreement for larger plasmon-photon coupling *g*_1_, smaller QE-plasmon coupling *g*_a_ and QE-photon coupling *g*_c_; but $\Delta \omega_{\text{0c}}^{2BS}$ (dashed lines) is less accurate for larger *g*_a_ and *g*_c_ at smaller *g*_1_. Furthermore, $\Delta \omega_{\text{0c}}^{2BS}$ is close to $\Delta \omega_{\text{0c}}^{\text{BS}}$ for small *g*_a_ and *g*_c_, and approaches $\Delta \omega_{\text{0c}}^{\text{BS}}$ as *g*_1_ increases, especially for the case of *g*_a_ = 5 meV and *g*_c_ = 0.4 meV as indicated in Figure 4a and b), where we can see that $\Delta \omega_{\text{0c}}^{2BS}\approx \Delta \omega_{\text{0c}}^{\text{BS}}$ for *g*_1_ > 30 meV. This suggests that it is possible to simultaneously access the single- and multi-atom qBS, and such hybrid cavities with the desirable level of plasmon-photon coupling have already been reported [4, 7]. In summary, the hybrid cavity can indeed be a versatile platform for diverse quantum-optics applications.

## 3 Conclusion and outlooks

With a quantized two-mode model for the LDOS of the plasmonic–photonic hybrid cavity, we unveil the formation of the atom-photon qBS due to the interference of different coupling pathways between QE and microcavity. Such dissipation-mediated light–matter interaction allows migrating the notion of Friedrich–Wintgen BIC into the plasmonic-photonic QED systems, leading to anomalously small decay for one of their eigenstates. Based on its small eigenenergy decay, we demonstrate two quantum-optics applications of atom-photon qBS with improved performance. Evidently, atom-photon qBS shows significant advantages in achieving high-efficiency single-photon generation and enhancing spontaneous entanglement generation. We also provide a generalized analytical condition of *N*-atom-photon qBS with *N* the number of QEs involved to help the future design of the experiments. To a larger extent, our work demonstrates a novel quantum-optics phenomenon in the hybrid cavity, which holds great potential for integrated optics, quantum optics, and quantum information applications. Nevertheless, it is worth noting that we consider the cryogenic temperature in this work, excluding the effect of pure dephasing. A brief discussion on the impact of pure dephasing on single-photon generation and spontaneous entanglement generation can be found in Supporting Information S5. Moving forward, we could extend the work by including more complex quantum effects, such as the synthetic magnetic flux [51, 52] and the environment-mediated Fano effect [53], and investigate more practical hybrid systems, for instance, those based on Mie nanoresonators [54, 55], and the strong coupling between plasmon and 2D transition metal dichalcogenide materials [56].

## Supplementary Material

Supplementary Material Details
