# Supplementary material for: Unveiling atom-photon quasi-bound states in hybrid plasmonic-photonic cavity
Source: Nanophotonics. 2022 Jun 9;11(14):3307–17. doi: 10.1515/nanoph-2022-0162 (PMC11502002; doi:10.1515/nanoph-2022-0162)
Supplement: Supplementary file 1 — Supplementary Material Details [file j_nanoph-2022-0162_suppl.pdf]

Supplementary

Yu-Wei Lu, Wen-Jie Zhou, Yongyao Li, Runhua Li, Jing-Feng Liu*, Lin Wu*, and Haishu Tan*

Unveiling atom-photon quasi-bound states in hybrid plasmonic-photonic cavity

S1 Analytical expression of the local density of states of plasmonic-photonic cavity

The quantum emitter (QE) dynamics in a cavity is governed by the local density of states (LDOS) or the spectral density, which can be numerically obtained via commercial softwares like Ansys/Lumerical FDTD solutions and COMSOL. The hybrid cavity constructs a structured environment that features a non-Lorentzian spectral density, which in general requires to be treated as continuous bosonic modes [1, 2] and are denoted as c_k in Fig. S1. On the other hand, in our model the hybrid cavity is composed of two coupled Lorentzian cavities, and hence its LDOS in principle can be generated using the parameters of two bare cavities and the coupling strength between them. To find an equivalent description of the LDOS of hybrid cavity that yields the same QE dynamics, we decompose the system in a non-Markovian core (QE-cavities and cavity-cavity interaction) and Markovian environment (cavities-bath interaction), as the schematic plotted in Fig. S1. The

Yu-Wei Lu, Yongyao Li, Haishu Tan, School of Physics and Optoelectronic Engineering, Foshan University, Foshan 528000, China. E-mail: tanhaishu@fosu.edu.cn
Wen-Jie Zhou, Lin Wu, Science, Mathematics and Technology (MT), Singapore University of Technology and Design (UTD), 8 Somapah Road, Singapore 487372.
***Corresponding author: Jing-Feng Liu,** College of Electronic Engineering, South China Agricultural University, Guangzhou 510642, China. E-mail:liujingfeng@scau.edu.cn
Runhua Li, School of Physics and Optoelectronics, South China University of Technology, Guangzhou 510641, China.
Yongyao Li, Guangdong-Hong Kong-Macao Joint Laboratory for Intelligent Micro-Nano Optoelectronic Technology, Foshan University, Foshan 528000, China.
***Corresponding author: Lin Wu,** Institute of High Performance Computing, Agency for Science, Technology, and Research (A*STAR), 1 Fusionopolis Way, #16-16 Connexis, Singapore 138632. E-mail:lin_wu@sutd.edu.sg

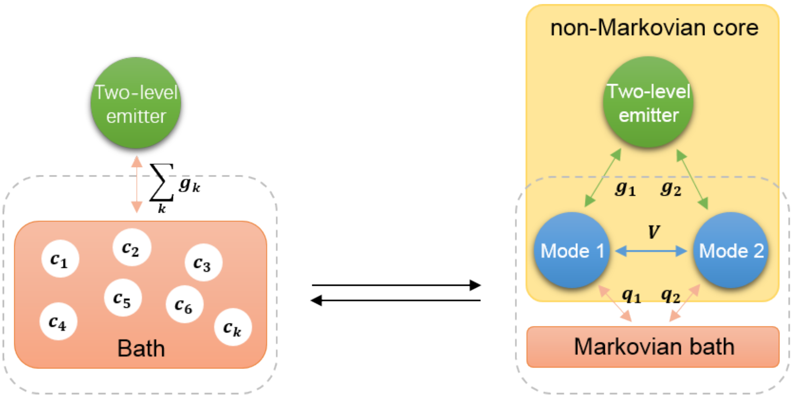

Fig. S1: Schematic of the mapping of the LDOS of a structured environment constituted of continuous bosonic modes c_k into a quantized two-mode model. The gray dashed lines indicate the structured environment. Base on the quantized two-mode model, the whole cavity QED system including the two-level QE can be decomposed into a non-Markovian core and the Markovian bath.

Hamiltonian of hybrid cavity reads

$$H' = H_0' + H_I' \tag{S1}$$

with the free Hamiltonian H_0' and the interaction Hamiltonian H_I'

$$H_0' = \omega_a a^\dagger a + \omega_c c^\dagger c + \sum_\mu \omega_\mu \alpha_\mu^\dagger \alpha_\mu \tag{S2}$$

$$H_I' = g_1 \left(a^\dagger c + c^\dagger a\right) + \sum_\mu \left[\left(V_\mu a^\dagger + U_\mu c^\dagger\right) \alpha_\mu + \alpha_\mu^\dagger \left(V_\mu^* a + U_\mu^* c\right)\right] \tag{S3}$$

where the Markovian environment is described by a bosonic reservoir, with α_μ and ω_μ being the annihilation operator and the frequency of μ-th mode, respectively. V_μ and U_μ are the corresponding coupling strengths to plasmonic antenna and microcavity, respectively. We can obtain the equations of motion for three fields

$$\dot{a} = -i\omega_a a - ig_1 c - i\sum_\mu V_\mu \alpha_\mu \tag{S4}$$

$$\dot{c} = -i\omega_c c - ig_1 a - i\sum_\mu U_\mu \alpha_\mu \tag{S5}$$

$$\dot{\alpha}_\mu = -i\omega_\mu \alpha_\mu - i\left(V_\mu^* a + U_\mu^* c\right) \tag{S6}$$

Formally integrating the equation for α_μ, we have

$$a_\mu = e^{-i\omega_\mu(t-t_0)}\alpha_\mu^0 - i\int_0^t d\tau e^{-i\omega_\mu(t-\tau)}\left(V_\mu^* a + U_\mu^* c\right) \tag{S7}$$

where a_μ^0 stands for the initial conditions at $t = 0$. The cavity-reservoir interaction follows the Markovian dynamics, thus it is sufficient to use the zero-order approximation $a(\tau) \approx a(t)e^{i\omega_a(t-\tau)}$ and $c(\tau) \approx c(t)e^{i\omega_c(t-\tau)}$. Substituting into Eq. (S7), we obtain

$$a_\mu = -i\int_0^t d\tau e^{i(\omega_\mu-\omega_a)(\tau-t)}V_\mu^* a - i\int_0^t d\tau e^{i(\omega_\mu-\omega_c)(\tau-t)}U_\mu^* c \tag{S8}$$

where we assume the initial condition $a_\mu^0 = 0$. The time integration yields a delta function and thus

$$a_\mu = -i\pi\delta\left(\omega_\mu - \omega_a\right)V_\mu^* a - i\pi\delta\left(\omega_\mu - \omega_c\right)U_\mu^* c \tag{S9}$$

Plugging back into Eqs. (S4) and (S5) we arrive at

$$\dot{a} = -i\left(\omega_a - \frac{i\kappa_a}{2}\right)a - i\left(g_1 - i\frac{\sqrt{\kappa_a^0\kappa_c}}{2}\right)c \tag{S10}$$

$$\dot{c} = -i\left(\omega_c - \frac{i\kappa_c}{2}\right)c - i\left(g_1 - i\frac{\sqrt{\kappa_a^0\kappa_c}}{2}\right)a \tag{S11}$$

where $\kappa_a^0 = 2\pi|V|^2$, $\kappa_c^0 = 2\pi|U|^2$ and we assume the response of reservoir is flat enough compared to the linewidth of cavities so that V and U are frequency independent and the subscript μ has been dropt. Note that κ_a^0 represents the radiative decay rate of cavity. The typical value of κ_a^0 for plasmonic antenna is several meV [3–5], and thus we can assume a real coupling strength between two cavities. The spectral density of hybrid cavity is given by [2, 6]

$$J(\omega) = \int_{-\infty}^{+\infty} d\tau \left\langle [g_a a(\tau) + g_c c(\tau)]\left[g_a a^\dagger(0) + g_c c^\dagger(0)\right]\right\rangle e^{i\omega t} \tag{S12}$$

where the two-time correlation functions $a(\tau)a^\dagger(0)$, $a(\tau)c^\dagger(0)$ and $c(\tau)c^\dagger(0)$ can be calculated using the quantum regression theorem with Eqs. (S10) and (S11) [7]. Then we can obtain the analytical expression of the spectral density of hybrid cavity using the independent parameters of two cavities and their coupling strength, which is given by

$$J(\omega) = -g_c^2\,\mathrm{Im}\left[J_c(\omega)\right] - 2g_a g_c\,\mathrm{Im}\left[J_{ac}(\omega)\right] - g_a^2\,\mathrm{Im}\left[J_a(\omega)\right] \tag{S13}$$

with

$$J_X(\omega) = \chi_X(\omega)\left[1 - g_1^2\chi_a(\omega)\chi_c(\omega)\right]^{-1} \tag{S14}$$

$$J_{ac}(\omega) = g_1\chi_a(\omega)\chi_c(\omega)\left[1 - g_1^2\chi_a(\omega)\chi_c(\omega)\right]^{-1} \tag{S15}$$

where $\chi_X(\omega) = [(\omega - \omega_X) + i\kappa_X/2]^{-1}$ for $X = a, c$. The above equations are equivalent to Eqs. (1)-(3) in the main text.

Fig. S2(a) shows the LDOS of hybrid cavity studied in the Fig. 1 in the main text for different resonance frequencies of plasmonic antenna ω_a. We can see that the dip of LDOS remains unchanged and is located at the resonance frequency of microcavity ω_c. For large plasmon-photon detuning $\Delta\omega_{ac}$, the LDOS peak around the microcavity resonance evolves into Fano lineshape. Fig. S2(b) plots the corresponding time evolution of initially excited QE with transition frequency $\omega_0 = \omega_c + \Delta\omega_{0c}^{BS}$, where $\Delta\omega_{0c}^{BS} \approx 0.77$meV. It shows that compared to the resonant case, large $\Delta\omega_{ac}$ leads to more obvious oscillation of QE dynamics, implying the stronger coherent interaction between the microcavity and the QE. From the LDOS we can see that it is the result of the formation of a sharp Fano peak in LDOS. This phenomenon, i.e., strong coherent interaction in $\Delta\omega_{ac} \neq 0$ instead of resonant plasmon-photon, is not contrary to the best performance of single-photon blockade achieves at $\Delta\omega_{ac} = 0$, because the QE dynamics is dominated by the LDOS around the transition frequency while the single-photon blockade depends on the eigenenergies structure of the QED system. Fig. S2(b) also compares the QE dynamics of hybrid cavity with a bare plasmonic antenna, where we can see the obviously prolonged lifetime of excited QE in hybrid cavity.

Fig. S2(c) studies the difference of the two-atom spontaneous emission (SE) spectra between the hybrid cavity and the bare components. It shows that there are symmetrical three peaks in the SE spectra of bare microcavity, while for bare plasmonic antenna only the central peak is existed due to the large dissipation. While the SE spectra of hybrid cavity presents a unique asymmetrical two-peak structure. The linewidth of the peak located at ω_0 is much narrower than another peak as well as that of bare components. Fig. S2(d) shows the corresponding SE dynamics of two atoms in hybrid cavity, where we can see that our two-mode model is valid and can obtain the correct time evolution and dynamical concurrence. We calculate the two-atom SE spectra and the corresponding SE dynamics following the theoretical framework and method in Ref. 8.

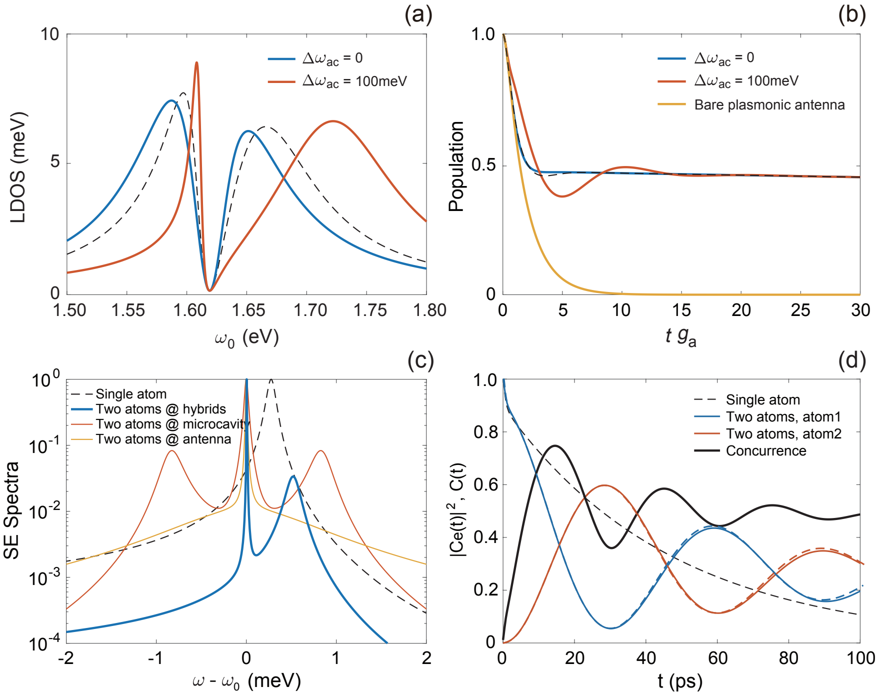

Fig. S2: (a) LDOS of hybrid cavity for various plasmon-photon detuning $\Delta\omega_{ac}$ by tuning the plasmon resonance. The black dashed line plots the LDOS of original structure shown in Fig. 1(b) in the main text. (b) The corresponding SE dynamics of QE. The result of a bare plasmonic antenna is shown for comparison. (c) Comparison of the normalized SE spectra of bare microcavity, bare plasmonic antenna, and hybrid cavity with $\omega_0 = \Delta\omega_{0c}^{BS}$. The cavities parameters are the same as Fig. 3 in the main text, while $\gamma = 0$. SE spectra of single atom in hybrid cavity is shown for comparison. (d) The corresponding SE dynamics in hybrid cavity and the concurrence of spontaneous entanglement generation. The results of numerical calculations (color dashed lines) and the SE dynamics of single atom (black dashed line) are shown for comparison.

S2 Analytical condition of atom-photon quasi-bound states and the decay of single-atom quasi-bound states

With the analytical expression of LDOS, the system parameters $(\omega_X, \kappa_X, g_X)$ of bare components can be obtained by fitting the LDOS curve of electromagnetic simulations with $-g_X^2 \mathrm{Im}\left[\chi_X(\omega)\right]$, where $X = a, c$. The mode coupling g_1 can be determined by second fitting with the previously obtained system parameters $(\omega_X, \kappa_X, g_X)$ using Eqs.(S13)-(S15). Then the dynamics of plasmonic-photonic QED system is described by the quantum master equation

$$\frac{\partial \rho}{\partial t} = i[\rho, H] + \frac{\kappa_a}{2}\mathcal{L}_a(\rho) + \frac{\kappa_c}{2}\mathcal{L}_c(\rho) + \sum_i \frac{\gamma}{2}\mathcal{L}_{\sigma_-^{(i)}}(\rho) \tag{S16}$$

with the Liouvillian superoperator

$$\mathcal{L}_{\hat{o}}(\rho) = 2\hat{o}\rho\hat{o}^\dagger - \hat{o}^\dagger\hat{o}\rho - \rho\hat{o}^\dagger\hat{o} \tag{S17}$$

and the two-mode Hamiltonian

$$\begin{aligned} H = {} & \omega_a a^\dagger a + \omega_c c^\dagger c + \sum_i \omega_0 \sigma_+^{(i)}\sigma_-^{(i)} + \sum_i g_a^{(i)}\left(a^\dagger\sigma_-^{(i)} + \sigma_+^{(i)} a\right) \\ & + \sum_i g_c\left(c^\dagger\sigma_-^{(i)} + \sigma_+^{(i)} c\right) - g_1\left(a^\dagger c + a c^\dagger\right) + H_{\text{drive}} \end{aligned} \tag{S18}$$

where H_{drive} is the driving Hamiltonian. In the weak-driving case, the system dynamics is well approximately by the jump-free part, therefore, we can rewrite Eq. (S18) as a non-Hermitian Hamiltonian

$$\begin{aligned} H' = {} & (\omega_a - i\kappa_a/2)\, a^\dagger a + (\omega_c - i\kappa_c/2)\, c^\dagger c + \sum_i (\omega_0 - i\gamma/2)\, \sigma_+^{(i)}\sigma_-^{(i)} \\ & + \sum_i g_a^{(i)}\left(a^\dagger\sigma_-^{(i)} + \sigma_+^{(i)} a\right) + \sum_i g_c\left(c^\dagger\sigma_-^{(i)} + \sigma_+^{(i)} c\right) - g_1\left(a^\dagger c + c^\dagger a\right) + H_{\text{drive}} \end{aligned} \tag{S19}$$

With the unitary transformation $U = \exp\left[-i\omega_c\left(c^\dagger c + a^\dagger a + \sigma_+\sigma_-\right)t\right]$ and resonant plasmon-photon interaction $\omega_a = \omega_c$, we can rewrite H' in the matrix

form

$$H'_N = \begin{pmatrix} -i\kappa_c/2 & -g_1 & g_c & g_c & \cdots & g_c & g_c \\ -g_1 & -i\kappa_a/2 & g_a^{(1)} & g_a^{(2)} & \cdots & g_a^{(N-1)} & g_a^{(N)} \\ g_c & g_a^{(1)} & \Delta\omega'_{0c} & 0 & \cdots & \cdots & 0 \\ g_c & g_a^{(2)} & 0 & \Delta\omega'_{0c} & \ddots & \cdots & 0 \\ \vdots & \vdots & \vdots & \ddots & \ddots & \ddots & \vdots \\ g_c & g_a^{(N-1)} & \vdots & \vdots & \ddots & \Delta\omega'_{0c} & 0 \\ g_c & g_a^{(N)} & 0 & 0 & \cdots & 0 & \Delta\omega'_{0c} \end{pmatrix} \tag{S20}$$

where $\Delta\omega'_{0c} = \Delta\omega_{0c} - i\gamma/2$. In the condition of $\kappa_a \gg \kappa_c, \gamma$, the genuine bound states with a purely real eigenvalue can form. The condition of bound states can be found by separating the real and imaginary parts of the characteristic polynomial of H'_N and setting to zero, which are given by

$$(-1)^{N+1} N^{N-2} g_c^{2(N-1)} \frac{\left(-Ng_1g_c + \lambda \sum_i g_a^{(i)}\right)^2}{\lambda^N} = 0 \tag{S21}$$

$$\Delta\omega_{0c} = \frac{\lambda^2 - g_c^2}{\lambda} \tag{S22}$$

where N is the number of QEs, λ stands for the eigenvalue, and we have used Eq. (S22) to eliminate $\Delta\omega_{0c}$ to obtain Eq. (S21). Eq. (S21) yields the analytical expression of N-atom bound states

$$\lambda_-^{nBS} \equiv \omega_c + \lambda = \omega_c + \frac{Ng_1g_c}{\sum_i g_a^{(i)}} \tag{S23}$$

The corresponding optimal QE-cavity detuning can be obtained from Eq. (S22)

$$\Delta\omega_{0c}^{nBS} = g_c \left(\frac{Ng_1}{\sum_i g_a^{(i)}} - \frac{\sum_i g_a^{(i)}}{g_1} \right) \tag{S24}$$

Particularly, for the single-atom case, we have

$$H'_1 = \begin{pmatrix} -i\kappa_c/2 & -g_1 & g_c \\ -g_1 & -i\kappa_a/2 & g_a \\ g_c & g_a & \Delta\omega_{0c} - i\gamma/2 \end{pmatrix} \tag{S25}$$

We can obtain the approximate expression of the imaginary part of λ_-^{BS} from H'_1, which is the decay of single-atom quasi-bound states (qBS)

$$\mathrm{Im}\left[\lambda_-^{BS}\right] \approx -\frac{\gamma}{2}\left[1 + \frac{\gamma}{\kappa_c}\frac{C_a\left(1 - \kappa_c/\gamma\right)}{C_1 + C_a\kappa_c/\gamma}\right]^{-1} \tag{S26}$$

where the cooperativity $C_a = g_a^2/\kappa_a\gamma$, $C_1 = g_1^2/\kappa_a\kappa_c$, and we have made the first-order expansion with respect to G_c, γ and κ_c. Eq. (S26) clearly indicates that the decay of atom-photon quasi-bound states is smaller than $\gamma/2$.

S3 Numerical calculations of averaged photon number and zero-time-delayed second-order correlation function

We apply a weak coherent pump to study the single-photon blockade effect, which belongs to the mechanism of conventional photon blockade [9]. The driving Hamiltonian H_{drive} takes the form of $H_{\text{drive}} = \Omega\left(e^{-i\omega_L t}\sigma_+ + \sigma_- e^{i\omega_L t}\right)$ for QE drive and $H_{\text{drive}} = \Omega\left(e^{-i\omega_L t}c^\dagger + ce^{i\omega_L t}\right)$ for cavity drive, where ω_L is the laser frequency of driving field. The averaged photon number $I_c = \left\langle c^\dagger c\right\rangle$ and the zero-time-delayed second-order correlation function $g^{(2)}(0) = \left\langle c^\dagger c^\dagger cc\right\rangle / I_c^2$ of the microcavity in hybrid cavity are obtained by numerically calculating the quantum master equation Eqs.(S16)-(S17) in the steady state using QuTip [10,11] with two-mode Hamiltonian Eq. (S18). The results of bare microcavity are obtained from the following quantum master equation

$$\frac{\partial\rho}{\partial t} = i\left[\rho, H_{SM}\right] + \frac{\kappa_c}{2}\mathcal{L}_c(\rho) + \frac{\gamma}{2}\mathcal{L}_{\sigma_-}(\rho) \tag{S27}$$

with the single-mode Hamiltonian

$$H_{SM} = \omega_c c^\dagger c + \omega_0\sigma_+\sigma_- + g_c\left(c^\dagger\sigma_- + \sigma_+ c\right) + H_{\text{drive}} \tag{S28}$$

By varying the QE-cavity detuning, the zero-time-delayed second-order correlation function of bare microcavity can be effectively tuned in the QE-driven case. The performance of single-photon blockade for plasmonic antenna is poor in the parameters range studied in this work and thus not shown here. In all calculations, the maximum photon number of bosonic operators a and c is 4.

Fig. S3 plots the I_c and $g^{(2)}(0)$ of the microcavity in hybrid cavity for QE drive. The single-photon blockade also reaches the best performance with simultaneously

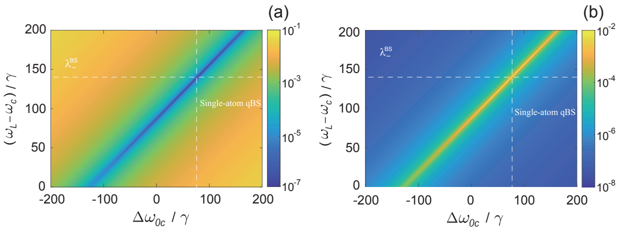

Fig. S3: Intensity I_c (a) and zero-time-delay correlation function $g^{(2)}(0)$ (b) in the QE driven case as the function of QE-cavity detuning $\Delta\omega_{0c}$ and the laser-cavity detuning, with ω_L being the laser frequency. The white dashed lines indicate the location of maximal intensity I_c or minimal $g^{(2)}(0)$. Parameters are the same as Fig. 2(a) in the main text.

highest single-photon purity and intensity at the single-atom quasi-bound state, which is the same as the case of cavity drive studied in the main text.

S4 Effective Hamiltonian and the spontaneous entanglement generation of two atoms in plasmonic-photonic cavity

As we discuss in the main text, the decay of two-atom quasi-bound states is minimal with identical atoms. The corresponding two-atom Hamiltonian reads

$$\begin{aligned} H_2 = \omega_a a^\dagger a + \omega_c c^\dagger c + \sum_{i=1,2} \omega_0 \sigma_+^{(i)} \sigma_-^{(i)} + \sum_{i=1,2} g_a \left(a^\dagger \sigma_-^{(i)} + \sigma_+^{(i)} a \right) \\ + \sum_{i=1,2} g_c \left(c^\dagger \sigma_-^{(i)} + \sigma_+^{(i)} c \right) - g_1 \left(a^\dagger c + a c^\dagger \right) \end{aligned} \tag{S29}$$

We can obtain the following equations of motion for atoms and cavities

$$\dot{a} = -i \left(\omega_a - \frac{i\kappa_a}{2} \right) a - i g_c \sum_{i=1,2} \sigma_-^{(i)} + i g_1 c \tag{S30}$$

$$\dot{c} = -i \left(\omega_c - \frac{i\kappa_c}{2} \right) c - i g_c \sum_{i=1,2} \sigma_-^{(i)} + i g_1 a \tag{S31}$$

$$\dot{\sigma}_-^{(i)} = -i \left(\omega_c + \Delta\omega_{0c} - \frac{i\gamma}{2} \right) \sigma_-^{(i)} - i g_a a - i g_c c \tag{S32}$$

where we have made the substitute $\omega_0 \equiv \omega_c + \Delta\omega_{0c}$. Since the plasmonic antenna is highly dissipative, we can formally integrate $\dot{a}$ and obtain in the resonant plasmonic-photonic coupling ($\omega_a = \omega_c$)

$$a \approx \frac{g_a \sum_{i=1,2} \sigma_-^{(i)}}{\Delta\omega_{0c} + \frac{i\kappa_a}{2}} - \frac{g_1 c}{\frac{i\kappa_a}{2}} \tag{S33}$$

Plugging back into Eqs. (S31) and (S32), we obtain

$$\dot{c} = -i \left[\omega_c - \frac{i\kappa_c}{2} \left(1 + \frac{4g_1^2}{\kappa_a \kappa_c} \right) \right] c - i \left(g_c - \frac{g_1 g_a}{\Delta\omega_{0c} + \frac{i\kappa_a}{2}} \right) \sum_{i=1,2} \sigma_-^{(i)} \tag{S34}$$

$$\begin{aligned} \sigma_-^{(i)} = -i \left[\omega_c + \Delta\omega_{0c} - \frac{i\gamma_0}{2} \left(1 + \frac{g_a^2}{\frac{\gamma_0}{2} \left(-i\Delta\omega_{0c} + \frac{K_a}{2} \right)} \right) \right] \sigma_-^{(i)} \\ -i \frac{g_a^2}{\Delta\omega_{0c} + \frac{i\kappa_a}{2}} \sigma_-^{(1+\delta_{1i})} - i \left(g_c - \frac{g_1 g_a}{\frac{i\kappa_a}{2}} \right) c \end{aligned} \tag{S35}$$

For $\Delta\omega_{0c}$ of atom-photon quasi-bound states is much smaller than $\kappa_a/2$, the obtained equations of motion are equivalent to a non-Hermitian effective Hamiltonian in single-excitation subspace

$$H_{\text{eff}} = \begin{pmatrix} \omega_c + \Delta\omega_{0c} - i\gamma_m & -i\gamma_p & g_{ca} \\ -i\gamma_p & \omega_c + \Delta\omega_{0c} - i\gamma_m & g_{ca} \\ g_{ac} & g_{ac} & \omega_c - i\kappa \end{pmatrix} \tag{S36}$$

where $\kappa = \kappa_c/2+\kappa_p$ and $\gamma_m = \gamma/2+\gamma_p$ with $\kappa_p = 2g_1^2/\kappa_a$ and $\gamma_p = 2g_a^2/(-i2\Delta\omega_{0c}+\kappa_a)$ being the plasmon-induced cavity and QE decays, respectively. $g_{ca} = g_c + i2g_ag_1/\kappa_a$ and $g_{ac} = g_c+i2g_ag_1/(-i2\Delta\omega_{0c}+\kappa_a)$ are the plasmon-mediated cavity-to-QE and QE-to-cavity couplings, respectively. Eq. (S36) can be written in a compact form

$$\begin{aligned} H_{\text{eff}} = \sum_{i,j=1,2} \left[\left(\omega_c + \Delta\omega_{0c} - i\frac{\gamma}{2}\right)\delta_{ij} - i\gamma_p\right]\sigma_+^{(i)}\sigma_-^{(j)} + (\omega_c - i\kappa)\,c^\dagger c \\ + \sum_{i=1,2}\left(g_{ac}c^\dagger\sigma_-^{(i)} + g_{ca}\sigma_+^{(i)}c\right) \end{aligned} \tag{S37}$$

The state vector of system with single excitation is given by

$$|\psi(t)\rangle = C_{eg}(t)|e\rangle_1|g\rangle_2|0\rangle_c + C_{ge}(t)|g\rangle_1|e\rangle_2|0\rangle_c + C_c(t)|g\rangle_1|g\rangle_2|1\rangle_c \tag{S38}$$

By solving the Schrödinger equation with initial conditions $C_{eg}(0) = 1$ and $C_{ge}(0) = C_c(0) = 0$, we can obtain the explicit expression of the probability amplitude $C_{eg}(t)$

$$\begin{aligned} C_{eg}(t) = \frac{e^{-\frac{i\Delta\omega_{0c}t}{2}t}e^{-i\omega_c t}}{2u}e^{-\frac{\Gamma_+}{2}t}\left[\frac{e^{ut/2} - e^{-ut/2}}{2}(-i\Delta\omega_{0c} + \Gamma_-)\right. \\ \left. + \left(\frac{e^{ut/2} + e^{-ut/2}}{2} + e^{\frac{-i\Delta\omega_{0c}+\Gamma_-}{2}t}\right)u\right] \end{aligned} \tag{S39}$$

where $u = \sqrt{-8g_{ca}g_{ac} + (-i\Delta\omega_{0c} + \Gamma_-)^2}$ and $\Gamma_\pm = \kappa\pm\gamma/2\pm2\gamma_p$. In the long-time approximation, we have $e^{ut/2} \gg e^{-ut/2}$, therefore,

$$C_{eg}(t) \approx \frac{e^{-\frac{i\Delta\omega_{0c}}{2}t}e^{-i\omega_c t}}{2}e^{-\frac{\Gamma_+}{2}t}\left(\frac{e^{\frac{ut}{2}}}{2}\frac{-i\Delta\omega_{0c} + \Gamma_- + u}{u} + e^{\frac{-i\Delta\omega_{0c}+\Gamma_-}{2}t}\right) \tag{S40}$$

Similarly, we can also obtain the approximate expression of $C_{ge}(t)$, which is required to calculate the concurrence

$$C_{ge}(t) \approx \frac{e^{\frac{i\Delta\omega_{0c}}{2}t}e^{i\omega_c t}}{2}e^{-\frac{\Gamma_+^*}{2}t}\left(\frac{e^{u^*t/2}}{2}\frac{i\Delta\omega_{0c} + \Gamma_-^* + u^*}{u^*} - e^{\frac{i\Delta\omega_{0c}+\Gamma_-^*}{2}t}\right) \tag{S41}$$

Therefore, $C_{eg}(t)C_{ge}^*(t)$ is given by

$$C_{eg}(t)C_{ge}^*(t) \approx \frac{e^{-(\kappa+\mathrm{Re}[\frac{\gamma}{2}+2\gamma_p])t}}{4}\left(\frac{e^{\mathrm{Re}[u]t}}{4}\frac{-i\Delta\omega_{0c}+\Gamma_-+u}{u}\frac{i\Delta\omega_{0c}+\Gamma_-^*+u^*}{u^*}\right.$$
$$+e^{\frac{-i\Delta\omega_{0c}+\kappa-\frac{\gamma}{2}+2\gamma_p}{2}t}\frac{e^{\frac{u^*t}{2}}}{2}\frac{i\Delta\omega_{0c}+\Gamma_-^*+u^*}{u^*}$$
$$\left.-e^{\frac{i\Delta\omega_{0c}+\kappa-\frac{\gamma}{2}+2\gamma_p^*}{2}t}\frac{e^{\frac{ut}{2}}}{2}\frac{-i\Delta\omega_{0c}+\Gamma_-+u}{u}-e^{(\kappa+\mathrm{Re}[-\frac{\gamma}{2}+2\gamma_p])t}\right) \tag{S42}$$

The above expression can be further simplified in consideration of $\kappa \gg \gamma_p \gg \gamma$, which is true for plasmonic-photonic cavity

$$4C_{eg}(t)C_{ge}^*(t) = \frac{e^{-\kappa-\gamma/2+\mathrm{Re}[u-2\gamma_p]t}}{4|u|^2}|\kappa-i\Delta\omega_{0c}+u|^2 - i\frac{e^{-\frac{\kappa+\frac{3\gamma}{2}}{2}t}e^{\frac{\mathrm{Re}[u-2\gamma_p]}{2}t}}{|u|^2}$$
$$\times\left[\sin\left(\frac{\mathrm{Im}\,[u-2\gamma_p]+\Delta\omega_{0c}}{2}t\right)\left(\kappa\,\mathrm{Re}[u]-\Delta\omega_{0c}\,\mathrm{Im}[u]+|u|^2\right)\right.$$
$$\left.-\cos\left(\frac{\mathrm{Im}\,[u-2\gamma_p]+\Delta\omega_{0c}}{2}t\right)(\kappa\,\mathrm{Im}[u]+\Delta\omega_{0c}\,\mathrm{Re}[u])\right]-e^{-\gamma t} \tag{S43}$$

We evaluate that $\mathrm{Im}\,[u-2\gamma_p]+\Delta\omega_{0c}\approx 0$ and $\mathrm{Re}[u]\gg\mathrm{Im}[u]$ for plasmonic-photonic cavity with moderate QE-plasmon coupling g_a, thus the final expression of dynamical concurrence $C(t)=2\left|C_{eg}(t)C_{ge}^*(t)\right|$ [12] is given by

$$C(t)\approx\frac{1}{2}\left|e^{-\gamma_- t}\frac{|\kappa-i\Delta\omega_{0c}+u|^2}{4|u|^2}-e^{-\gamma t}\right.$$
$$\left.-ie^{-\frac{\gamma}{2}t}e^{-\frac{\gamma_-}{2}t}\left(\frac{\kappa\,\mathrm{Re}[u]}{|u|^2}+1\right)\sin\left(\frac{\mathrm{Im}\,[u-2\gamma_\mathrm{p}]+\Delta\omega_{0c}}{2}t\right)\right| \tag{S44}$$

where $\gamma_-=\kappa+\gamma/2-\mathrm{Re}\,[u-2\gamma_p]$. The lower bound of $C(t)$ is given by the real part of the terms inside the absolute value sign of Eq. (S44), while the upper bound is obtained by setting $\sin\left[(\mathrm{Im}\,[u-2\gamma_p]+\Delta\omega_{0c})\,t/2\right]=1$ in Eq. (S44).

Fig. S4(a) gives the dynamical concurrence $C(t)$ with the same parameters as Fig.3(b) in the main text, but for $\Delta\omega_{0c}=0$. It shows that the analytical expression Eq. (S44) is also valid in this case. Fig. S4(b)-(d) compare the location of minimum $|\gamma_-|/\gamma$ with the two-atom qBS $\Delta\omega_{0c}^{2BS}$, where we can see the good accordance between them for different coupling parameters. Fig. S4(b)-(d) also shows that the impact of QE-photon coupling g_c on the minimum $|\gamma_-|/\gamma$ is negligible, while the large QE-plasmon coupling g_a greatly increases the value of minimum $|\gamma_-|/\gamma$, and can change the sign of $\Delta\omega_{0c}^{2BS}$. Furthermore, large g_1 has positive effect on reducing the minimum $|\gamma_-|/\gamma$. Since the minimum $|\gamma_-|/\gamma$ determines the damping of the oscillation in $C(t)$, it is related to the maximum value of $C(t)$. Therefore,

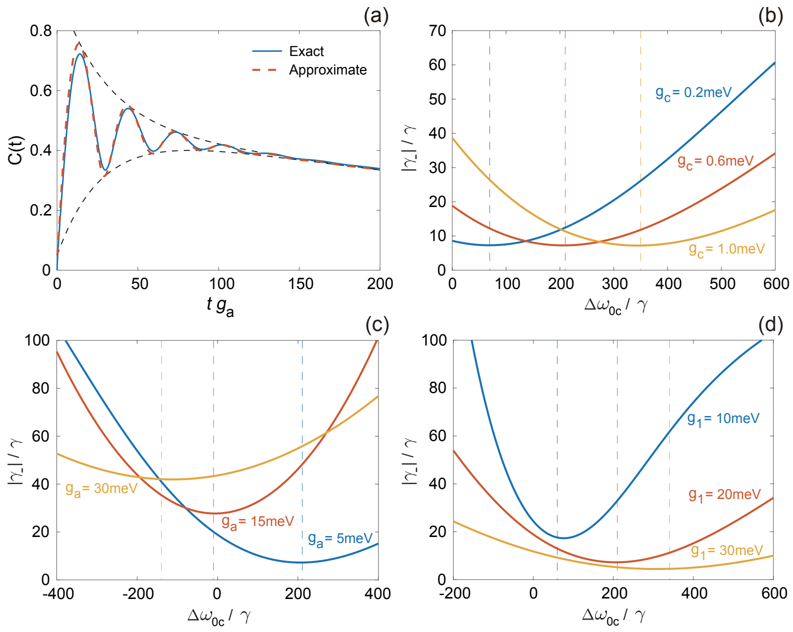

Fig. S4: (a) Comparison of the analytical expression Eq. (S44) and the numerically calculated dynamical concurrence with $\Delta\omega_{0c} = 0$. The dashed black lines give the upper and lower bounds of dynamical concurrence. (b)-(d) $|\gamma_-|/\gamma$ for various coupling parameters. The vertical lines with the same color indicate the condition of two-atom bound states given by Eq. (S24). Other parameters of hybrid cavity are the same as Fig. 3 in the main text unless specially noted.

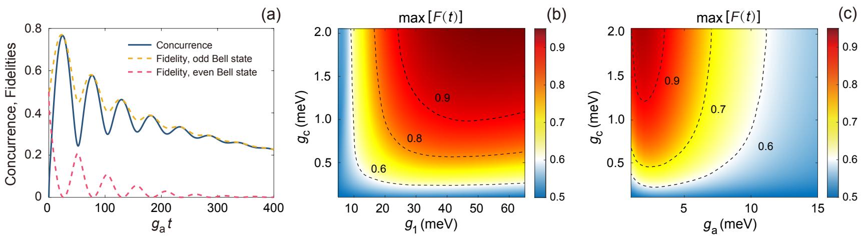

Fig. S5: (a) Dynamical concurrence and fidelity for odd and even Bell states. The parameters are the same as the inset of Fig. 3(b) in the main text. (b) and (c) Maximum fidelity $\max[F(t)]$ of $|\Psi_-\rangle$ as the function of g_c and g_1, g_c and g_a, respectively. The parameters are the same as Figs. 3(e) and (f) in the main text.

Fig. S4(b)-(d) indicate that a large g_1 is beneficial to achieve high concurrence of spontaneous entanglement generation (SEG), while a large g_a is undesirable. Note that g_c determines the achievable maximum $C(t)$, and strong QE-photon coupling plays important role in enhancing the SEG, as shown in Figs. 3(e) and (f) in the main text.

To better understand the generated entanglement in hybrid cavity, we calculate the fidelity $F(t) = \langle\Psi_i|\rho(t)|\Psi_i\rangle$ against different entangled states, with $|\Psi_\pm\rangle = (|eg\rangle \otimes |00\rangle \pm |ge\rangle \otimes |00\rangle)/\sqrt{2}$ being the even ($i = +$) and odd ($i = -$) Bell states, where $|eg\rangle/|ge\rangle$ represents the state that the first/second QE is in the excited state while another is in the ground state, and $|00\rangle$ is the vacuum state of cavity fields. The results are shown in Fig. S5(a), where we can see that the entangled state created is the odd Bell state $|\Psi_-\rangle$. Fig. S5(b) and (c) plot the maximum fidelity of $|\Psi_-\rangle$ as the function of g_c and g_1, g_c and g_a, respectively. By comparing with Figs. 3(e) and (f) in the main text, we can see that $\max[F(t)] \approx \max[C(t)]$.

S5 Impact of pure dephasing on single-photon generation and spontaneous entanglement generation

In the above discussion and in the main text, the effect of pure dephasing is not included, since we consider the cryogenic temperature. The pure dephasing of QE can be introduced by implementing a dissipator in the quantum master equation

$$\frac{\partial\rho}{\partial t} = i[\rho, H] + \frac{\kappa_a}{2}\mathcal{L}_a(\rho) + \frac{\kappa_c}{2}\mathcal{L}_c(\rho) + \sum_i \frac{\gamma}{2}\mathcal{L}_{\sigma_-^{(i)}}(\rho) + \sum_i \frac{\gamma_n}{2} D_{\sigma_z^{(i)}}(\rho) \qquad \text{(S45)}$$

where γ_n is the dephasing rate, and $D_{\sigma_z}(\rho) = \sigma_z\rho\sigma_z - \rho$ is the dissipator for pure dephasing of QE. Fig. S6 (a) and (b) show the I_c and $g^{(2)}(0)$ with various γ_n for cavity drive and QE drive, respectively. It shows that the minimum $g^{(2)}(0)$ (maximum I_c) decreases by nearly three orders of magnitude for $\gamma_n = 0.1$meV (10γ). However, the impact of pure dephasing outside the dip and peak region is not obvious for both cavity and QE drive. Increasing γ_n to 1meV (100γ), the dip (peak) of $g^{(2)}(0)$ (intensity) is hard to recognize. Therefore, the low-temperature environment is still required for obtaining better performance of single-photon generation even utilizing the single-atom qBS.

On the contrary, the spontaneous entanglement generation manifests robustness against the weak and even moderate pure dephasing. As shown in Fig. S6 (c) and (d), both concurrence and fidelity are less affected by the pure dephasing even γ_n reaches 100γ. The negative impact of pure dephasing is significant for $\gamma_n = 10$meV,

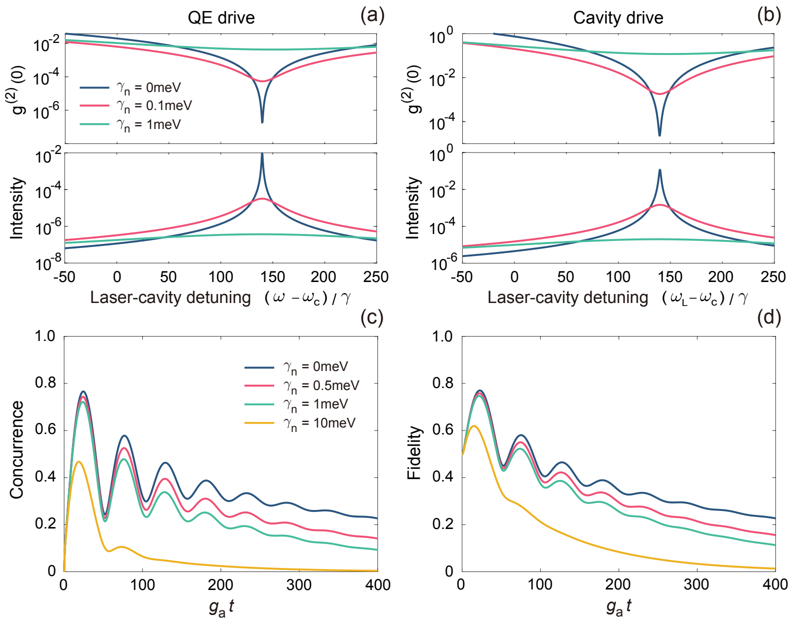

Fig. S6: (a) and (b) Impact of pure dephasing on single-photon generation for cavity drive and QE drive, respectively. Other parameters are the same as the inset of Fig. 3(b) in the main text. (c) and (d) Impact of pure dephasing on concurrence and fidelity of odd Bell state $|\Psi_-\rangle$, respectively. Other parameters are the same as Fig. S5 (a).

which is comparable to the typical linewidth of QE at ambient conditions [13, 14]. Therefore, two-atom qBS is promising in realizing room-temperature quantum entanglement.

References

[1] Ho Trung Dung, Ludwig Knöll, and Dirk-Gunnar Welsch. Spontaneous decay in the presence of dispersing and absorbing bodies: General theory and application to a spherical cavity. *Physical Review A*, 62(5):053804, 2000.

[2] D. Tamascelli, A. Smirne, S. F. Huelga, and M. B. Plenio. Nonperturbative treatment of non-markovian dynamics of open quantum systems. *Physical Review Letters*, 120(3):030402, 2018.

[3] Hugo M. Doeleman, Ewold Verhagen, and A. Femius Koenderink. An-

tenna–cavity hybrids: Matching polar opposites for purcell enhancements at any linewidth. *ACS Photonics*, 3(10):1943–1951, 2016.

[4] P. Peng, Y. C. Liu, D. Xu, Q. T. Cao, G. Lu, Q. Gong, and Y. F. Xiao. Enhancing coherent light-matter interactions through microcavity-engineered plasmonic resonances. *Physical Review Letters*, 119(23):233901, 2017.

[5] Mohsen Kamandar Dezfouli, Reuven Gordon, and Stephen Hughes. Modal theory of modified spontaneous emission of a quantum emitter in a hybrid plasmonic photonic-crystal cavity system. *Physical Review A*, 95(1):013846, 2017.

[6] Emil V. Denning, Jake Iles-Smith, and Jesper Mork. Quantum light-matter interaction and controlled phonon scattering in a photonic fano cavity. *Physical Review B*, 100(21):214306, 2019.

[7] Marlan O Scully and M Suhail Zubairy. *Quantum optics*. Cambridge University Press, 1999.

[8] Ho Trung Dung, Ludwig Knöll, and Dirk-Gunnar Welsch. Resonant dipole-dipole interaction in the presence of dispersing and absorbing surroundings. *Physical Review A*, 66(6), 2002.

[9] Eduardo Zubizarreta Casalengua, Juan Camilo López Carreño, Fabrice P. Laussy, and Elena del Valle. Conventional and unconventional photon statistics. *Laser & Photonics Reviews*, 14(n/a):1900279, 2020.

[10] J. R. Johansson, P. D. Nation, and Franco Nori. Qutip: An open-source python framework for the dynamics of open quantum systems. *Computer Physics Communications*, 183(8):1760–1772, 2012.

[11] J. R. Johansson, P. D. Nation, and Franco Nori. Qutip 2: A python framework for the dynamics of open quantum systems. *Computer Physics Communications*, 184(4):1234–1240, 2013.

[12] William K. Wootters. Entanglement of formation of an arbitrary state of two qubits. *Physical Review Letters*, 80(10):2245–2248, 1998.

[13] Haijun Qiao, Keith A. Abel, Frank C. J. M. van Veggel, and Jeff F. Young. Exciton thermalization and state broadening contributions to the photoluminescence of colloidal pbse quantum dot films from 295 to 4.5 k. *Physical Review B*, 82(16):165435, 2010.

[14] Mohammad Ramezani, Alexei Halpin, Antonio I. Fernández-Domínguez, Johannes Feist, Said Rahimzadeh-Kalaleh Rodriguez, Francisco J. Garcia-Vidal, and Jaime Gómez Rivas. Plasmon-exciton-polariton lasing. *Optica*, 4(1), 2016.
